# Validation of stool and blood analysis compared to *Inv A* and *ttr* based direct blood qPCR assay as diagnostic tools for typhoid fever

**DOI:** 10.1186/s12866-025-04127-9

**Published:** 2025-07-05

**Authors:** Shymaa A. Elaskary, Hanem Mohamed Badawy, Doaa S. Elgendy, Amany Mohammed Abdelmaksoud, Rasha G. Mostafa

**Affiliations:** 1 Medical Microbiology and Immunology Department, Faculty of Medicine, Menoufia University, Shebin al-Kom, Egypt; 2 Clinical Pathology Department, Faculty of Medicine, Menoufia University, Shebin al-Kom, Egypt; 3 Internal Medicine Department, Faculty of Medicine, Menoufia University, Shebin al-Kom, Egypt

**Keywords:** Typhoid fever, *Salmonella* infections, Stool microbiology, Blood microbiology, Polymerase chain reaction (PCR), *Tetrathionate respiration* genes & *Inv A* gene detection

## Abstract

**Background:**

The development of molecular techniques, such as polymerase chain reaction (qPCR), provides a promising approach for detecting *S. enterica* due to its rapidity, sensitivity, specificity, and resistance to interference from antibiotics, surpassing the routinely used stool and blood culture methods.

**Aim:**

This study aims to compare different stool and blood culturing methods with the direct blood qPCR-based assay of *Salmonella Invasion A* (*Inv A*) and *Salmonella Tetrathionate Respiration* (*ttr*) virulence genes as diagnostic tools for typhoid fever.

**Method:**

One hundred clinically diagnosed typhoid fever (CDTF) cases were subjected to conventional stool and blood culture, followed by standard biochemical and serotyping identification of *S. enterica*, compared to direct blood and enriched blood-based *ttr* and *Inv A* qPCR-based techniques.

**Results:**

Using the enriched blood-based *ttr* and *Inv A* qPCR technique, 90% and 85% of cases, respectively, were positive and confirmed as typhoid fever. While the blood-based *ttr* and *Inv A* qPCR technique showed 82% and 80% positivity, respectively, with a Ct threshold value of 37 cycles for both techniques. The stool culture method revealed *S. enterica* infection in only 32% of cases, while blood culture methods identified *S. enterica* in 48% of cases.

**Conclusion:**

The enriched blood-based *ttr* and *Inv A* qPCR method is a promising rapid tool for diagnosing typhoid fever, offering high sensitivity, specificity, accuracy and unaffected by antibiotic intake.

## Background

*Salmonella enterica* encompasses human-restricted pathogens, *Salmonella typhi* (*S. typhi*) and *Salmonella paratyphi* (*S. paratyphi*) types *A*, *B*, and *C*, which are linked to the onset of typhoid fever [1]. Most human salmonellosis cases are foodborne. However, infections can also be contracted through direct or indirect contact with animals. In high-income countries, enhanced food and water sanitation practices have nearly eradicated this infection. However, in less developed areas of Asia and Africa, the infection remains endemic, leading to approximately 20 million cases and 200,000 deaths annually [2].

The traditional clinical indicator for diagnosing typhoid fever is a progressive increase in fever and toxicity, resembling a step ladder pattern. However, these symptoms can be obscured by improper use of antibiotics. Therefore, it is crucial to have an accurate laboratory diagnosis of typhoid fever, especially when atypical symptoms are present, to distinguish it from other febrile illnesses [1].

The standard method for investigating enteric fever is through bacterial cultures obtained from bone marrow aspiration that exhibit high sensitivity, above 90%, and are less affected by previous antibiotic therapy. However, this invasive method is not suitable for routine use [3].

Blood and stool cultures are the most common methods for diagnosing *Salmonella* infections in humans [4]. Cultures derived from blood specimens yielded a higher number of *Salmonella typhi* isolates compared to those from conventional stool cultures [4].

Accurate laboratory diagnosis of typhoid fever is crucial for the prompt administration of antibiotics, which helps to reduce both mortality and morbidity [5].

The development of molecular techniques, such as polymerase chain reaction (PCR), offers a promising approach for detecting *S. enterica* due to its rapidity, sensitivity, specificity, and resistance to interference from antibiotics [6].

The virulence genes of *Salmonella* are located within large genomic regions called *Salmonella* Pathogenicity Islands (SPIs). These SPIs are recognized by their distinctive guanine and cytosine content compared to the core genome. They are often associated with specific tRNA genes and mobile genetic elements such as insertion sequences, transposons, or bacteriophage genes [7].

A recent study has demonstrated that the qPCR method exhibits good sensitivity and specificity for detecting the *Inv A* and *ttr* genes of *Salmonella* [8].The *Inv A* gene is located within SPI1, which facilitates the pathogen’s internalization into human epithelial cells [9].The *ttr* gene is found in SPI 2 and encodes tetrathionate reductase, which plays a role in the bacterium’s life cycle [10].There is a lack of sufficient data regarding the use of molecular methods for diagnosing enteric fever in Egypt that considered the golden standard test [1].

This study aims to evaluate various stool and blood culturing methods in comparison to direct blood qPCR-based detection of *Inv A* and *ttr Salmonella* virulence genes as diagnostic tools for typhoid fever.

## Methods

This cross-sectional study included one hundred adults who were clinically diagnosed with typhoid fever (CDTF). These patients are presented with stepwise increasing fever, accompanied by abdominal pain, diarrhea, or constipation. The participants were recruited from outpatient clinics and various inpatient wards at Menoufia University Hospitals between January 2024 and February 2025. In total, 100 blood samples and 100 stool samples were collected from all participants following standard laboratory procedures and promptly sent to the Medical Microbiology and Immunology Department at Menoufia University Faculty of Medicine regardless of any prior antibiotic intake.

### Stool samples

One gram of each fresh fecal specimen was aseptically transferred into a sterile test tube containing 9 ml of 0.1% sterile buffered peptone water (BPW, ISO-CM1049) to prepare stool homogenate, which was incubated at 37 °C for 20 h as a pre-enrichment step [11, 12].

One ml of the pre-enrichment stool homogenate was then inoculated into a 10 ml sterile Selenite broth (SB) test tube and incubated at 37 °C for selective enrichment (BD Diagnostics, Franklin Lakes, NJ, USA). A 10 µl loopful of the incubated broth was streaked onto Xylose Lysine Deoxycholate agar (XLD) at 37 °C for 24 h to isolate, identify, and purify *Salmonella* for further biochemical testing [13].

### Blood samples

Ten milliliters of blood were obtained from each participant using an aseptic technique. Seven ml were inoculated into a BacT/Alert blood culture bottle (bioMérieux, USA) and immediately loaded into the instrument. Positive blood cultures were subcultured on blood agar and MacConkey agar at 37 °C for one day. The result was considered negative if no growth was observed after 7 days of incubation [5, 14].

The remaining 3 ml of blood were divided equally into three parts: one part was added to plain tube to separate serum for Widal test (Omega, UK) where the significant Widal test titre for diagnosing typhoid fever is generally considered to be ≥ 1:160 for *Salmonella typhi* O and H antigens. Another part was kept untreated, and the last one was pre-enriched with an equal volume of TSB containing 5% bile (bioMérieux, USA) and incubated for 5 h at 37 °C. Both pre-enriched and untreated blood samples were stored at −20 °C for further qPCR analysis [14].

### Ethical consideration

The study received approval from the Menoufia Faculty of Medicine Ethical Committee (2/2025MICR4) and written consent was obtained from each participant.

### Morphological and conventional biochemical identification

Initial identification of isolates was performed using Gram staining and oxidase tests. Isolates that were Gram-negative and oxidase-negative were further tested using conventional biochemical reactions, including triple sugar iron agar, lysine iron agar, motility indole ornithine, citrate utilization, and urease tests (Oxoid) [15].

### *Salmonella* single tube; acidic lysine Tryptophan Iron motility agar (ALTIMA)

The ALTIMA tube includes six biochemical tests: glucose fermentation, lysine decarboxylation (LDC), tryptophan deamination (TDA), indole production, H₂S production, and motility testing. A single, well-isolated colony (non-lactose fermenting and H₂S positive from the selective medium) was picked with a straight needle loop and stabbed into the *Salmonella* single tube, then incubated at 37 °C for 24 h. The medium changes to black with a yellow surface layer upon adding Kovac’s reagent, indicating the presence of *Salmonella species* [16].

### Serological identification of *Salmonella* isolates

All biochemically confirmed *Salmonella* isolates were serologically identified using the Kauffman-White scheme by slide agglutination with Remel agglutinating sera (ThermoFisher, USA) according to the manufacturer’s protocol [17].

### Real time PCR for detection of *Salmonella* invasion gene A (*Inv A*) and *Salmonella* tetrathionate respiration gene (*ttr*)

#### DNA extraction

DNA was extracted from blood samples and TSB bile-enriched blood using the QIAamp DNA extraction kit (Qiagen, Germany) as per the manufacturer’s instructions. The extracted DNA was stored at −20 °C until it was ready for qPCR analysis.

#### Real -time PCR protocol

A real-time PCR system (Applied Biosystems 7500, ThermoFisher, USA) was utilized for amplifying and detecting the amplified products. The reaction mix for each primer was prepared separately in a well, reaching a total volume of 25 µl, as follows: 12.5 µl of Maxima SYBR Green qPCR Master Mix, 1 µl of forward primer (20 µM), 1 µl of reverse primer (20 µM), 5 µl of *Salmonella* DNA, and 5.5 µl of nuclease free water.

As controls, 5 µl of extracted DNA from a pure *Salmonella* culture served as a positive control, and 5 µl of sterile nuclease free water was used as a negative control. The qPCR process included an initial denaturation at 95˚C for 10 min for one cycle, followed by three-step cycling for 60 cycles: denaturation at 95˚C for 15 s, annealing at 60˚C for 30 s, and extension at 72˚C for 30 s. Melting curve analysis of the PCR products was performed to confirm their specificity and identity. Fluorescence was measured using the qPCR instrument software. Samples with Cycle threshold (Ct) values of less than 37 cycles were considered positive. The primer sequences used were as follows [8]:

- *ttr*: F:5’-CTCACCAGGAGATTACAACATGG-3‘(94 bp), R: AGCTCAGACCAAAAGTGACCATC3’,
- *Inv A*: F:5’-AGCGTACTGGAAAGGGAAAG-3‘(284 bp), R:5’CACCGAAATACCGCCAATAAAG-3’,

#### Statistical analysis

The data were collected, tabulated, and statistically analyzed using IBM’s Statistical Package for the Social Sciences (SPSS) version 23 (SPSS Inc., Chicago, IL, USA). Descriptive statistics were applied, where quantitative data were presented as mean, standard deviation (SD), median, and range, and qualitative data were presented as numbers and percentages. The tests of significance included the Chi-square test (χ2) to examine the association between qualitative variables. The Kappa test assessed agreement between different diagnostic tests. The validity of each diagnostic test was evaluated by calculating sensitivity, specificity, positive predictive value, negative predictive value, and accuracy. The student’s t-test and the Mann-Whitney test were used to assess differences between two sets of normally distributed and binomial distributed quantitative data, respectively. A P value greater than 0.05 was considered non-significant, while a P value less than 0.05 was considered significant.

## Results

The study involved 100 patients who were clinically diagnosed with typhoid fever (CDTF) based on clinical examination and stepwise fever patterns. The group consisted of 62 males and 38 females, with an average age of 29.82 ± 12.04 years. The majority (65%) of the patients resided in rural areas. Among the symptoms observed, 45% had fever accompanied by diarrhea, 47% had fever with abdominal pain, and 10% had fever with constipation, as detailed in Table 1.

**Table 1:** Demographic characteristics and clinical profiles of the studied cases

Demographic data	The studied cases *N* = 100	
Age (years)		
Mean ± SD	29.82 ± 12.04	
Range	14–55	
Sex		
Male	62	62.0
Female	38	38.0
Residence		
Rural	65	65.0
Urban	35	35.0
Clinical presentation		
Abdominal pain	47	47.0
Diarrhea	45	45.0
Constipation	10	10.0

*SD* Standard Deviation

Culturing stool samples on XLD agar revealed the presence of *S. enterica* in 32% of cases, with 68% showing no growth, demonstrating a highly statistically significant difference (*P* < 0.001). Serotyping indicated that *S. typhi* was the predominant serotype (90.6%), followed by *S. paratyphi A* (6.2%) and *S. paratyphi B* (3.2%), with a highly significant difference (*P* < 0.001). Blood cultures identified *S. enterica* in 48% of cases, with 52% showing no growth and no statistically significant difference (*P* = 0.57). The serotyping of blood isolates showed 83.3% *S. typhi*, 10.4% *S. paratyphi A*, and 6.3% *S. paratyphi B*, with a highly significant difference (*P* < 0.001). Widal testing was positive in 75% of cases, with antibody titters to the O antigen above 1/80, showing 81.3% *S. typhi*, 13.3% *S. paratyphi A*, and 5.4% *S. paratyphi B*, with a highly significant difference (*P* < 0.001) as detailed in Table 2.

**Table 2:** Phenotypic analysis of stool and blood samples for detection of *S. enterica* in the studied cases

Phenotypic analysis methods	The studied cases *N* = 100					
	Positive	Negative	S. enterica Serotypes			
			*S. typhi*	*S. Paratyphi A*	*S. Paratyphi B*	Test (*P* value)
**Stool culture**	32 (32%)	68 (68%)	29 (90.6%)	2 (6.2%)	1 (3.2%)	4.9 (< 0.001)^1^ 6.5 (< 0.001)^2^
**Blood analysis**						
- Blood culture	48 (48%)	52 (52%)	40 (83.3%)	5 (10.4%)	3 (6.3%)	0.42 (0.57)^1^ 7.4(< 0.001)^2^
- Widal testing	75 (75%)	25 (25%)	61 (81.3%)	10 (13.3%)	4 (5.4%)	6.9 (< 0.001)^1^ 9.2 (< 0.001)^2^
Combined stool & blood culture positive	32 (32%)	68 (68%)	29 (90.6%)	2 (6.2%)	1 (3.2%)	4.9 (< 0.001)^1^ 6.5 (< 0.001)^2^

*Salmonella* single tube testing (ALTIMA)	Conventional biochemical tests				
	XLD		Blood culture		
	Sensitivity (100%), specificity (100%), PPV (100%), NPV (100%), accuracy (100%).				
	Stool WBCs				
	< 50/HPF		50–100/HPF	> 100/HPF	Test (*P* value)
Positive stool culture *N* = 32 (32%)	2 (6.25%)		10 (31.25%)	20(62.5%)	1.39 (0.17)^3^ 2.36 (0.02)^4^ 2.73 (0.002)^5^
Negative stool culture *N* = 68 (68%)	13 (19.1%)		40 (58.8%)	15 (22.1%)	

1: Comparing between positive and negative cases, 2: Comparing different salmonella serotypes, 3: Comparing positive and negative stool culture (stool WBCs < 50/HPF), 4: Comparing positive and negative stool culture (stool WBCs 50–100/HPF), 5: Comparing positive and negative stool culture (stool WBCs > 100/HPF)

The used *Salmonella* single tube ALTIMA test showed 100% sensitivity, specificity, PPV, NPV and accuracy regarding different used conventional biochemical reactions across the different culture methods as detailed in Table 2.

In *Salmonella*-positive stool samples, approximately 62.5% have a high percentage of stool white blood cells (WBCs) exceeding 100/HPF with statistically significant difference with negative stool cultures (*P* < 0.02). Also, around 58.8% of *Salmonella*-negative stool samples exhibit 50 to 100 WBCs/HPF with highly statistically significant difference (*P* < 0.002) regarding *Salmonella*-positive stool samples as detailed in Table 2.

The XLD stool culture method showed 84% agreement and accuracy (kappa = 0.675) with blood culture that considered the reference culture method with sensitivity, specificity, PPV and NPV of 66.7%,100%, 100% and 76.5%, respectively and high statistically significant difference (*P* < 0.001) as detailed in Table 3.

**Table 3:** Comparative effectiveness of stool culture against blood culture for detecting *S. enterica*

	Blood culture		Kappa	*P* value	Agreement
Stool culture	Positive (48)	Negative (52)			
Positive (32) Negative (68)	32 (66.7) 16 (33.3)	0 (0.0) 52 (100)	0.675	< 0.001	84%
	Sensitivity Specificity	66.7% 100%	PPV NPV	100% 76.5%	Accuracy 84%

PPV = Positive predictive value, NPV = negative predictive value

**Fig. 1:**
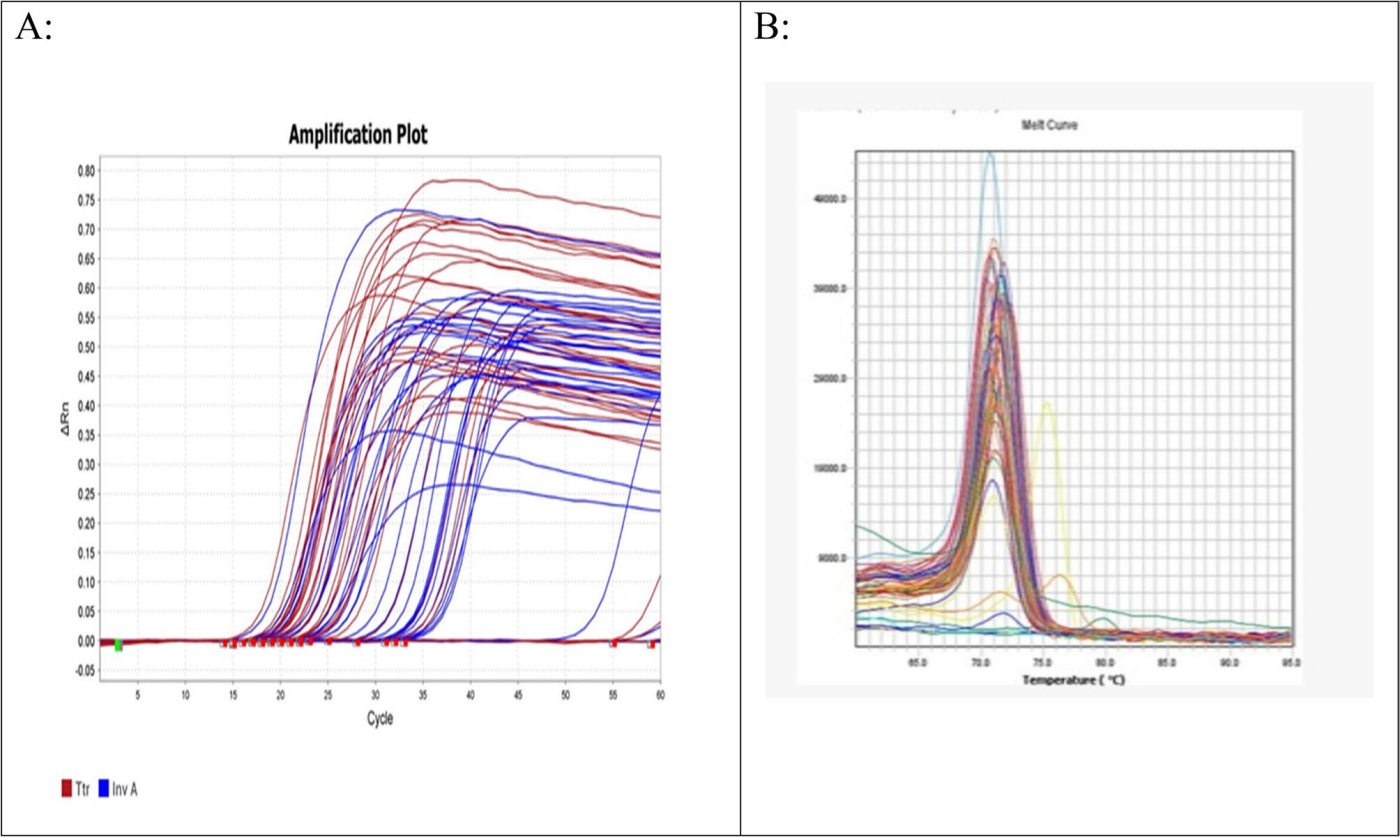
**A** Amplification plot of *ttr* and *Inv A* genes versus cycle number of enriched blood samples,** B** Melting plot curve for both *ttr* and *Inv A* genes

For molecular analysis, the blood-based *ttr* gene qPCR method demonstrated that 82 of the CDTF were positive and 18 were negative while the enriched blood *ttr* gene qPCR method showed that 90 CDTF were positive and only ten of them were negative. The blood-based *ttr* gene qPCR demonstrated an accuracy and agreement rate of 92% (kappa = 0.672) compared to the gold standard enriched blood-based *ttr* gene qPCR method for identifying *S. enterica*, showing a highly statistically significant difference (*P* < 0.001). This method achieved 91.1% sensitivity, 100% specificity, 100% positive predictive value (PPV), and 55.6% negative predictive value (NPV). In contrast, the blood-based *Inv A* gene qPCR detection method showed 95% accuracy and agreement (kappa = 0.83) with the enriched blood-based *Inv A* qPCR detection, with a highly statistically significant difference (*P* < 0.001). This method achieved 94.1% sensitivity, 100% specificity, 100% PPV, and 75% NPV for detecting *S. enterica* were detected only 80 CDTF as positive as detailed in Table 4; Fig. 1A, B.

**Table 4:** Evaluating Blood-based qPCR versus enriched Blood-based qPCR for detecting *S. enterica Inv A* and *ttr* diagnostic genes

Molecular analysis	Enriched blood- based qPCR				
	ttr gene				
**Blood- based qPCR**	Positive (90) 90%	Negative (10)10%	**Kappa**	*P* ** value**	**Agreement**
*ttr* gene					
Positive (82) 82% Negative (18) 18%	82 (91.1) 8 (8.9)	0 (0.0) 10 (100)	0.672	< 0.001	92%
	Sensitivity Specificity	91.1% 100%	PPV NPV	100% 55.6%	Accuracy 92%

	***Inv A *** **gene**				
	Positive (85) 85%	Negative (15) 15%	**Kappa**	*P* value	**Agreement**
*Inv A* gene					
Positive (80) 80% Negative (20) 20%	80 (94.1) 5 (5.9)	0 (0.0) 15 (100)	0.83	< 0.001	95%
	Sensitivity Specificity	94.1% 100%	PPV NPV	100% 75%	Accuracy 95%

*PPV* Positive predictive value, *NPV* negative predictive value

By comparing the different culture methods for detecting *S. enterica* against the gold standard enriched blood-based *ttr* gene qPCR detection method, the XLD stool culture showed 42% agreement and accuracy (Kappa = 0.1) with 35.6% sensitivity, 100% specificity, 100% positive predictive value (PPV) and 14.7% negative predictive value (NPV). The blood culture results demonstrated 58% agreement and accuracy, (Kappa = 0.19) with 53.3% sensitivity, 100% specificity, 100% PPV and 19.2% NPV as detailed in Table 5; Fig. 1A, B.

**Table 5:** Comparative evaluation of different cultures methods against golden standard enriched Blood-based *ttr* gene qPCR for detecting *S. enterica*

Culture methods	Enriched blood base ttr gene qPCR				
	Positive (90)	Negative (10)	Kappa	*P* value	Agreement
**Stool culture**					
Positive Negative	32 (35.6) 58 (64.4)	0 (0.0) 10 (100)	0.10	0.02	42%
	Sensitivity Specificity	35.6% 100%	PPV NPV	100% 14.7%	Accuracy 42%
**Blood culture**					
Positive Negative	48 (53.3) 42 (46.7)	0 (0.0) 10 (100)	0.19	0.001	58%
	Sensitivity Specificity	53.3% 100%	PPV NPV	100% 19.2%	Accuracy 58%

*PPV* Positive predictive value, *NPV* Negative predictive value

**Fig. 2:**
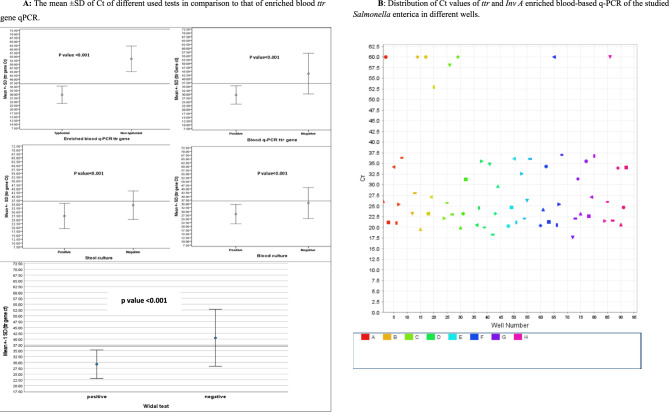
**A** The mean ± SD of Ct of different used tests in comparison to that of enriched blood *ttr* gene qPCR. **B** Distribution of Ct values of *ttr* and *Inv A* enriched blood-based q-PCR of the studied *Salmonella* enterica in different wells. The Ct cutoff value of ≤ 37 determining positive test by q-PCR. The values near to 37 as 36 or 35 are considered low positive and values far from 35 as 25, 22, 20 are considered highly positive. On the other hand, values higher than 37 are considered negative samples as Ct 60

The distribution of Ct values in the 100 CDTF cases using the enriched blood *ttr* gene qPCR method, the gold standard, is presented in Table 6; Fig. 2A & B, with 37 Ct as the cutoff value. Among the 90 typhoidal cases, Ct ranged from 17.5 to 37, with a mean ± SD of 29.64 ± 5.77 and a median of 30.5 Ct. In contrast, the 10 nontyphoidal cases had a Ct range of 38 to 60, a mean ± SD of 53.55 ± 8.51, and a median of 57.25 Ct, showing a highly significant difference (*P* < 0.001). Amplification signals at high Ct values in nontyphoidal cases were likely due to background noise, non-specific amplification, contamination, cross-reactivity, or instrumental artifacts. The positively detected *ttr* gene had a Ct range of 17.5 to 37 (mean ± SD: 29.54 ± 6.01, median: 30.5 Ct), while negative cases ranged from 28 to 60 (mean ± SD: 43.42 ± 13.26, median: 38.5 Ct) with a statistically significant difference (*P* < 0.001). For stool cultures, positive cases had a Ct range of 18 to 37 (mean ± SD: 26.41 ± 5.85, median: 26 Ct), whereas negatives ranged from 17.5 to 60 (mean ± SD: 34.68 ± 9.63, median: 33.25 Ct) with a significant difference (*P* < 0.001). In blood cultures, positives ranged from 17.5 to 37 (mean ± SD: 28.20 ± 6.45, median: 29 Ct), while negatives varied from 19 to 60 (mean ± SD: 35.58 ± 10.33, median: 33.25 Ct, *P* < 0.001). Regarding Widal testing, positive cases had a Ct range of 17.5 to 37 (mean ± SD: 29.2 ± 6.1, median: 30 Ct), while total negatives ranged from 28 to 60 (mean ± SD: 40.54 ± 12.2, median: 35 Ct), showing a highly significant difference (*P* < 0.001).

**Table 6:** Cycle threshold values of the gold standard, enriched blood-based *ttr* qPCR in comparison with different used diagnostic tests

ttr gene	Positive *N* = 90								Negative *N* = 10	Test	*P* value
Ct Mean ± SD Median Range	29.64 ± 5.77 30.5 17.5–37								53.55 ± 8.51 57.25 38–60	11.80	< 0.001
	Stool culture		Blood culture		Widal test		Blood ttr qPCR				
	**Positive** *N* = 32	**Negative** *N* = 58	**Positive** *N* = 48	**Negative** *N* = 42	**Positive** *N* = 75	**Negative** *N* = 15	**Positive** *N* = 82	**Negative** *N* = 8	**Non typhoid** *N* = 10	Test	*P* value
**Ct**										4.33	< 0.001^1^
Mean ± SD Median Range	26.41 ± 5.85 26.0 18–37	31.43 ± 4.93 33.0 17.5–37	28.20 ± 6.45 29.0 17.5–37	31.30 ± 4.41 32.0 19–37	29.2 ± 6.1 30 17.5–37	31.86 ± 2.9 32 28–37	29.54 ± 6.01 30.5 17.5–37	30.75 ± 2.0 30.75 28–33.5	53.55 ± 8.51 57.25 38–60	2.69 2.58 0.06	0.02^2^ 0.01^3^ 0.96^4^

1: Comparing stool culture positive and stool culture negative

2: Comparing blood culture positive and blood culture negative

3: Comparing Widal positive and Widal negative

4: Comparing Blood *ttr* qPCR positive and negative

**Fig. 3:**
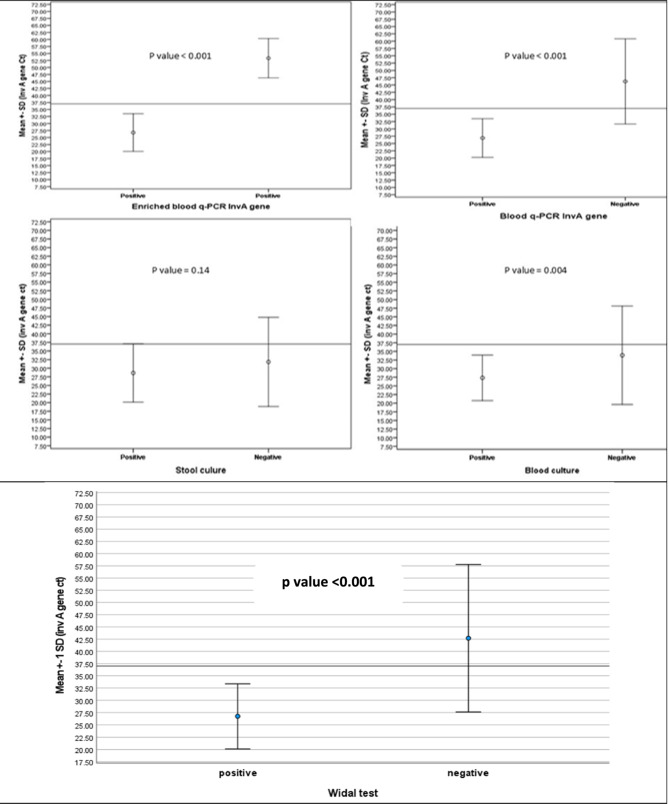
The mean ± SD of Ct of different used tests in comparison to that of enriched blood *Inv A* gene qPCR

The enriched blood *Inv A* gene qPCR method (Table 7; Fig. 3) identified 85 typhoidal cases with a Ct range of 17.5–37 (mean ± SD: 26.75 ± 6.72, median: 26 Ct), while 15 negative cases showed Ct values between 38 and 60 (mean ± SD: 53.30 ± 7.04, median: 55.5 Ct), with high statistical significance (*P* < 0.001). Blood qPCR for *Inv A* produced Ct values of 17.5–37 (mean ± SD: 26.86 ± 6.59, median: 26 Ct) in typhoidal cases, whereas negatives ranged from 18 to 60 (mean ± SD: 46.23 ± 14.58, median: 50.75 Ct), again showing significant differentiation (*P* < 0.001). Stool cultures positives had a Ct range of 19–37 (mean ± SD: 27.22 ± 6.07, median: 26.5 Ct), while negatives varied from 17.5 to 60 (mean ± SD: 32.39 ± 13.24, median: 31 Ct, *P* = 0.14, non-significant). Blood cultures positive cases showed a Ct range of 17.5–37 (mean ± SD: 27.33 ± 6.58, median: 27 Ct), while negatives ranged 18–60 (mean ± SD: 33.87 ± 14.26, median: 32.75 Ct, *P* = 0.004, statistically significant). Widal testing positives had Ct values 17.5–37 (mean ± SD: 26.75 ± 6.6, median: 25 Ct), while negatives ranged 28–60 (mean ± SD: 42.7 ± 15.07, median: 47 Ct) with a highly significant difference (*P* < 0.001).

**Table 7:** Cycle threshold values of the enriched blood-based *Inv A* qPCR in comparison with different used diagnostic tests

Inv A	Positive *N* = 85								Negative *N* = 15	Test	*P* value
Ct Mean ± SD Median Range	26.75 ± 6.72 26 17.5–37								53.30 ± 7.04 55.5 38–60	14.01	< 0.001
	Stool culture		Blood culture		Widal test		Blood qPCR ttr gene				
	**Positive** *N* = 32	**Negative** *N* = 53	**Positive** *N* = 48	**Negative** *N* = 37	**Positive** *N* = 75	**Negative** *N* = 10	**Positive** *N* = 80	**Negative** *N* = 5	**Non typhoid** *N* = 15	Test	*P* value
Ct										0.49	0.62^1^
Mean ± SD Median Range	27.22 ± 6.07 26.5 19–37	26.47 ± 7.12 24.0 17.5–37	27.33 ± 6.58 27.0 17.5–37	26.0 ± 6.91 23.0 18–37	26.75 ± 6.6 25 17.5–37	26.8 ± 7.78 28 18–37	26.86 ± 6.59 26 17.5–37	25.0 ± 9.25 19 18–37	53.30 ± 7.04 55.5 38–60	0.91 0.17 0.89	0.37^2^ 0.86^3^ 0.37^4^

1: Comparing stool culture positive and stool culture negative

2: Comparing blood culture positive and blood culture negative

3: Comparing Widal positive and Widal negative

4: comparing Blood qPCR *Inv A* gene positive and negative

## Discussion

Typhoid fever remains a significant public health issue in less developed countries. This study aims to demonstrate and clarify the most reliable and rapid diagnostic test. Stool and blood samples were collected from 100 clinically diagnosed typhoid fever cases (62 males and 38 females) with an average age of 29.82 ± 12.04 years, all of whom attended Menoufia University Hospitals during the study period. All cases experienced step ladder fever accompanied by diarrhea (45%), abdominal pain (47%), or constipation (10%). These clinical presentations are consistent with findings from other studies [18, 19]. Most cases (65%) were from rural areas, similar to previous study that confirmed the linkage of *S. enterica* infection with inadequate sanitation and health education [20].

In our study, stool culture shows 84% agreement and accuracy (kappa = 0.675) with blood culture for detecting *Salmonella* isolates with 66.7% sensitivity, 100%specificity, 100% PPV and 76.5% NPV. Stool cultures identified *S. enterica* in 32% of the cases. While Blood culturing methods detected *S. enterica* in 48% of the cases studied with positive Widal testing in 75% of the cases. This finds agreement with Qassim et al. [4] and others [21, 22] who documented that the culture from blood was found to be more sensitive than the culture from stool. And recommended it as gold standard culture method for *S. typhi* isolation.

However, Maddocks et al. and Ruiz Gomez et al. reported lower rates of *Salmonella* positive cases by 1.8%, and 11.3% respectively [23, 24].

*S. typhi* was the most isolated species, accounting for 90.6% of positive stool isolates, while *S. paratyphi A* and *S. paratyphi B* were detected in 6.2% and 3.2% of cases, respectively. Similar results were observed in blood culture positive isolates, with *S. typhi* representing 81.3%, and *S. paratyphi A* and *S. paratyphi B* making up 13.3% and 5.4%, respectively. These findings are in line with numerous previous studies [25–27].

Our study demonstrated that the *Salmonella* single tube method had 100% reliability, matching the accuracy of traditional biochemical tests. This method also shows potential for screening lactose-negative stool isolates, which may indicate the presence of *Salmonella*. Its applicability is especially valuable in highly endemic areas, as described by Procop et al. [28].

In general, Mahmoud et al. [1] demonstrated 51% sensitivity, 100% specificity and 75.5 accuracy of blood culture for *Salmonella* detection regarding the clinical diagnosis with 70% positivity by using Widal testing. In the same context, previous studies have reported blood culture sensitivity ranging from 40 to 75% [29, 30]. The reduced sensitivity for diagnosing *S. enterica* can be attributed to the effects of antibiotic therapy and the reduction of bacteremia in the later stages of infection [30].

Our results demonstrated that the enriched blood-based qPCR detection method for *ttr* and *Inv A* genes outperformed the traditional blood-based qPCR approach, exhibiting higher sensitivity and specificity in alignment with previous studies. This enhanced performance is likely attributed to the higher concentration of target genes, which minimizes false negatives and improves detection thresholds [1, 8].

Numerous studies have assessed various *Salmonella* -specific genes for molecular detection, with *ttr* and *Inv A* being among the most utilized. Research indicates that the *ttr* gene demonstrates high specificity for *Salmonella enterica*, especially in environmental and clinical specimens. Likewise, the *Inv A* gene, a crucial virulence factor, has been widely validated for its exceptional sensitivity in identifying *Salmonella* across diverse sample types [31].

Although *ttr* and *Inv A* have shown strong reliability in detecting *Salmonella*, additional molecular targets, including *bcfD*, *phoP*, and *siiA*, have been investigated for identification purposes [31]. However, research suggests that primers targeting *ttr* offer greater inclusivity and consistent amplification, making them the preferred choice for clinical diagnostics and food safety applications [32].

In our study the Ct cut off value in qPCR detection of *ttr* and *Inv A* genes in both blood and enriched blood-based methods was 37cycles that agreed with Das et al. [5] using the *Flic- d* gene qPCR method and differ from Mahmoud et al. [1] who documented that Ct cut off value < 35 cycles for *Inv A* and *ttr* genes.

Ct (Cycle Threshold) values play a vital role in assessing both the presence and quantity of the target genetic material. A lower Ct value signifies a higher concentration of the target gene, whereas a higher Ct value indicates a reduced amount of genetic material [33].

In this study the results of blood-based qPCR, stool culture and blood culture methods were verified and compared to the enriched blood based *ttr* and *Inv A* qPCR, where the cases showed Ct equal, or less than 37 cycles diagnosed as typhoidal cases and those showed Ct more than 37 cycles diagnosed as nontyphoidal. This is in accordance with previous studies [5, 34, 35].

The findings of Nair et al. were consistent with our results, demonstrating that Ct values below 37 cycles were strongly linked to typhoidal strains, while higher Ct values corresponded to lower bacterial loads or nontyphoidal infections. These observations reinforce prior research emphasizing the significance of establishing a reliable Ct threshold for precise diagnosis [36].

In our research, we demonstrated the Ct value in detail comparing the results between different used test while this specific topic in Egypt remains limited, particularly regarding the detection of target genes in human blood samples. Our study provides a more comprehensive and detailed investigation, offering valuable insights that enhance the understanding of *Salmonella* molecular detection.

## Conclusions

Delayed and inadequate diagnosis of *Salmonella enterica* has significantly contributed to the emergence of highly virulent, multidrug-resistant strains, emphasizing the urgent need for rapid and reliable diagnostic methods. This issue is particularly critical in developing regions where typhoid fever remains endemic, and conventional detection methods often lack the sensitivity required for early and accurate identification. The enriched blood-based *ttr* and *Inv A* qPCR method demonstrates high sensitivity and specificity, making it a valuable molecular tool for improved pathogen detection. By providing rapid and precise results, it minimizes reliance on traditional culture methods, which can be time-consuming and affected by prior antibiotic use, leading to false negatives. Its findings contribute to the growing body of research advocating for the standardization of qPCR-based techniques, enhancing diagnostic reliability and guiding future studies aimed at controlling typhoid fever in endemic areas.

## Data Availability

All data used to support the findings of this study are available from the corresponding author on request.

## Abbreviations

CDTF: Clinically diagnosed typhoid fever
Inv A: Salmonella Invasion A
ttr: Salmonella Tetrathionate Respiration
qPCR: Quantitative polymerase chain reaction
SPI1,2: Salmonella pathogenicity island 1, 2
XLD: Xylose Lysine Deoxycholate agar
ALTIMA: Salmonella single tube; Acidic Lysine Tryptophan Iron Motility Agar
